# Real-World Evidence from the Integrative Medicine Primary Care Trial (IMPACT): Assessing Patient-Reported Outcomes at Baseline and 12-Month Follow-Up

**DOI:** 10.1155/2019/8595409

**Published:** 2019-06-26

**Authors:** Robert L. Crocker, Jason T. Hurwitz, Amy J. Grizzle, Ivo Abraham, Rick Rehfeld, Randy Horwitz, Andrew T. Weil, Victoria Maizes

**Affiliations:** ^1^Andrew Weil Center for Integrative Medicine, College of Medicine, University of Arizona, Tucson, AZ, USA; ^2^Center for Health Outcomes & PharmacoEconomic Research (HOPE), College of Pharmacy, University of Arizona, Tucson, AZ, USA

## Abstract

**Purpose:**

The University of Arizona Integrative Health Center (UAIHC) was an innovative membership-supported integrative medicine (IM) adult primary care clinic in Phoenix, Arizona. UAIHC delivered healthcare using an integrative medicine model that combined conventional and complementary medical treatments, including nutrition, mind-body medicine, acupuncture, manual medicine, health coaching, educational classes, and groups. Results from pre-post evaluation of patient-reported outcomes on several standardized measures are presented here.

**Methods:**

UAIHC patients completed surveys at baseline and after 12 months of continuous integrative primary care. Patients reported on perceived changes in health outcomes as measured by* Short-Form Health Survey* (*SF-12* general, mental, and physical health),* Perceived Stress Scale (PSS4)*,* Work Productivity and Activity Impairment Questionnaire (WPAI)*,* World Health Organization Well-Being Index (WHO-5*),* Pain Visual Analog Scale (VAS)*,* Fatigue Severity Scale (VAS; FSS)*,* Generalized Anxiety Disorder Scale (GAD2)*,* Patient Health Questionnaire* for depression* (PHQ2), Pittsburgh Sleep Quality Index (PSQI) *global rating of sleep quality, and the* Behavioral Risk Factor Surveillance System (BRFSS*; nutrition, exercise, and physical activity). Overall differences between time points were assessed for statistical significance. Patient demographics are also described.

**Results:**

177 patients completed baseline and follow-up outcome measures. Patients were predominantly white, female, college-educated, and employed. Baseline to one-year follow-up results indicate statistically significant improvements (*p <*.05) on all but perceived stress (*PSS-4*) and work absenteeism (*WPAI*). Clinical impact and/or practical effects are reported as percent change or standardized effect sizes whenever possible. Other demographic and descriptive information is summarized.

**Conclusions:**

Following one year of IM primary care at UAIHC, patient-reported outcomes indicated positive impacts in several areas of patients' lives: mental, physical, and overall health; work productivity; sleep quality; pain; fatigue; overall well-being; and physical activity.

## 1. Introduction

The University of Arizona Integrative Health Center (UAIHC) was an innovative membership-supported integrative medicine (IM) adult primary care clinic in Phoenix, Arizona. UAIHC used a hybrid payment model to deliver comprehensive holistic healthcare combining conventional and complementary medical treatments. The Integrative Medicine Primary Care Trial (IMPACT) evaluates the impact of integrative primary care on patients' health using the model of care delivered at the UAIHC. The IMPACT study design is described elsewhere [1–3], but summarized briefly here to contextualize the results reported herein. Considering the integrative primary care model as the intervention, the IMPACT study addresses three high level questions: (1) Was integrative primary care delivered true to the design (fidelity of the intervention)? (2) Did the intervention have an impact on patients' health and well-being as measured by standardized patient-reported outcome measures? (3) Did the intervention impact other outcomes, including cost and utilization? The* first* question regarding intervention fidelity was addressed previously [3] and is summarized below. An analysis of employer health claims data is underway to answer the* third* question regarding cost and utilization outcomes. This paper addresses the* second* IMPACT study question on whether the care received at UAIHC impacted patients' health and well-being. These results derive from a pre-post comparison of patient-reported outcomes on multiple standardized measures.

## 2. Background

Integrative medicine (IM) is patient-centered, whole person care that emphasizes the body's innate healing capacity and the importance of lifestyle to enhance health [4, 5]. IM is an evidence-based, prevention-oriented, clinical approach that* integrates* conventional medical approaches with evidence-supported complementary medicine (CM) modalities [6, 7].

The University of Arizona Integrative Health Center (UAIHC), in Phoenix, Arizona, was an innovative clinic that delivered integrative primary care via a patient-centered, team-based model [1, 3]. UAIHC was staffed by 2 fulltime University of Arizona Andrew Weil Center for Integrative Medicine (AWCIM) IM fellowship-trained primary care physicians and by complementary practitioners including a chiropractor, an acupuncturist, a behavioral health clinician, a dietitian, a health coach and a nurse. Prior to opening the UAIHC, all staff members completed a 2-week training period providing a shared foundation in IM, introductory sessions in Motivational Interviewing and Mindfulness-Based Stress Reduction, and an in-depth review of current literature on integrative and complementary approaches to the treatment of common chronic conditions. The UAIHC model used a hybrid financing structure that combined health insurance reimbursement with membership fees paid by patients and/or employers.

Groups and classes were available to patients on topics such as nutrition, stress reduction, optimal weight and lifestyle, in addition to yoga and Tai Chi. During almost 4 years of operation, 1,700 patients received care at the clinic.

Since the integrative primary care model was the intervention, it was important to first establish its fidelity, (i.e., whether or not the intervention was delivered as intended [8]). As measured by patient-reported experiences, the IM care provided at UAIHC was consistent with the practice model design. Patients felt that they received whole person care, established positive caring relationships with providers who promoted their self-care and well-being, and reported high overall satisfaction with UAIHC [3]. Having established the fidelity of the intervention, the results presented here address whether or not the intervention had an impact on patients' health and well-being assessed by standardized patient-reported outcome measures. As outlined in the published protocol [2] five sets of analyses were planned for the IMPACT study: (1) demographics and baseline values of the first 200 patients enrolled; (2) fidelity assessment of the clinic operations; (3) semistructured patient interviews for qualitative assessment of the clinic experience; (4) changes in health outcomes from baseline to one year; and (5) economic analysis utilizing insurance claims. This paper focuses on the fourth analysis, a prospective cohort study of the changes in health-related outcomes, with the primary outcome measure being health-related quality of life.

## 3. Methods

### 3.1. Participants

Following approval by the University of Arizona institutional review board, the UAIHC invited all patients to complete baseline questionnaires after their first visit and again after 12-months of continuous enrollment. Upon enrollment in the study, participants received a hyperlink to access the baseline questionnaire on a secure system. Patients received another hyperlink to a follow-up questionnaire just prior to 12 months of continual enrollment. For each of these questionnaires, the system automatically generated secure email reminders to nonresponders at 3, 5, and 10 days following the initial contact for each of these questionnaires. Although this process was automated, the study coordinator would follow up with nonresponders to encourage them to complete the questionnaire. This study was designed as a real-world observational study of daily clinical practice, with visits scheduled per clinicians' best clinical judgment; hence patients did not necessarily have scheduled follow-up visits at specific time intervals. In accordance with the protocol [2], this paper reports on the subset of UAIHC patients who received services for at least 12 months and for whom we had both baseline and 12-month questionnaire responses since this was the most complete data set available for analysis.

### 3.2. Measures

Patients reported on health outcomes using several brief, published, standardized, and some norm-referenced measures summarized in Table 1. Patients completed measures at baseline and 12 months.

### 3.3. Data Preparation

Data used for analyses contained no direct identifying patient information and were stored on secured servers at the University of Arizona. Random, unique patient identification codes were used to link and merge the separate baseline and follow-up data sets. Data were examined for missing, illogical, or out-of-range values.

Patients who completed baseline and 12-month assessments for at least one outcome measure were included in the analyses. Scoring required complete data for each measure on each time point, resulting in different sample sizes for the various outcomes. Publicly available algorithms were used to score the measures.

### 3.4. Analyses

Study participants served as their own control; baseline values were compared to those measured after 12 months. Overall differences between time points were assessed using the paired (dependent)* t-*test or—when parametric test assumptions were not met—using the nonparametric alternative Wilcoxon Signed Rank test. All two-tailed* p*-values less than .05 were considered statistically significant. Confidence intervals of differences for* t-*tests and effect sizes for both tests are also reported [21]. Medians, as well as means, are reported for all nonparametric test results.

## 4. Results

A total of 253 patients completed 12 months in the study. Of these, 76 were excluded due to missing either baseline or month 12 outcomes measures, resulting in a 70% participation rate.  Table 2 summarizes demographic information about the 177 UAIHC patients included in this study. Overall, most patients were highly educated (86% completed a 4-year college degree or more), employed (75%), Caucasian (86%), and female (70%). Most patients were married or lived with a partner (69%) and over 27% reported an annual gross family income ≥$150,000; 58% self-paid for their UAIHC membership without employer contribution. The most commonly cited reasons for seeking healthcare at UAIHC were to prevent a serious health problem (34%) and to maximize patient's health whether or not their illness was curable (23%). Average age was 52 years old (SD = 10.8,* n* = 159).

Patients (*N* = 177) completed follow-up questionnaires at 12.7 months on average (SD = 0.9) from baseline. Results of the published standardized measures are summarized in Table 3. Statistically significant improvements were observed on all but one of the measures (i.e.,* PSS-4*).

### 4.1. Health-Related Quality of Life

At 12 months the* SF-12* showed statistically significant improvements on the average* General Health Item *scores (*p *= .002,* r *= .17), the* Physical Component Summary* scores, (*p* = .002,* r *= .24, 95% CI 0.80, 3.55), and the* Mental Component Summary* scores (*p* < .001,* r *= .29, 95% CI 1.51, 4.68).

### 4.2. Stress

The improvement in overall perceived stress scores on the* PSS-4* (0 to 16) was not statistically significant (*p *= .08). However, an additional two-item measure (*Stress Response and Amount*) indicated statistically significant improvement in participants' amount of stress in life over the past year and their ability to handle stress (*p *< .001,* r *= .33).

### 4.3. Work Productivity

For employed patients (62% of respondents) reporting on the past seven days using the* WPAI*, the percent of time missed at work (absenteeism) did not differ from baseline (*Mdn *< 0.01%) to 12 months (*Mdn* < 0.01%*; p *= .110,* r* = -.11). However, there was a significant decrease in the percent of time impaired while working (presenteeism) from baseline (*Mdn *= 15%) to 12 months (*Mdn *= 10%;* p *= .003,* r *= -.21). Overall work impairment due to health (i.e., absenteeism and presenteeism combined) was significantly lower at 12-months (*Mdn* = 10%) than at baseline (*Mdn* = 20%;* p *= .002,* r *= -.22). For all respondents, employed and unemployed, overall activity impairment due to health over the past seven days was significantly lower at 12 months (*Mdn* = 10%) than at baseline (*Mdn *= 20%;* p *= .019,* r *= -.13).

### 4.4. Mental Health

On average, patients reported a significant improvement in overall well-being on the* WHO-5* from baseline to 12 months (*p *< .001,* r *= .34, 95% CI 3.79, 9.30). A statistically significant decrease in average* GAD-2 *anxiety summary scores was also observed (*p* < .001), representing a moderate effect (*r* = 0.27). In addition, the* PHQ-2* depression summary scores indicated a statistically significant improvement (*p* = .002), representing a modest effect (*r* = 0.24).

### 4.5. Sleep

Global ratings of sleep quality significantly improved (*p* < .001) as measured on the* PSQI*. Patients were more likely to rate good quality sleep (i.e., fairly good or very good) at 12 months (74%) than at baseline (60%).

### 4.6. Nutrition and Physical Activity

The BRFSS indicated increased consumption of vegetables (*p *< .01,* r* = -0.17), but no difference in fruits consumed. Patients also reported a significant increase in the average number of times per week engaged in their primary physical activity (*p* < .01,* r* = -0.17). The number of times per week patients engaged in muscle strengthening activities also increased significantly (*p* < .05).

### 4.7. Subgroups Reporting Specific Conditions

#### 4.7.1. Pain

Among 65 respondents who initially reported that pain was a frequent problem, a statistically significant decrease in pain ratings on the Pain Visual Analog Scale was found (*p *< .001), representing a moderate effect (*r *= 0.48).

#### 4.7.2. Fatigue

Likewise, among 94 patients who initially reported that fatigue or tiredness were frequent problems, a statistically significant decrease in fatigue ratings on the Fatigue Visual Analog Scale was also observed (*p* < .001), representing a large effect (*r* = 0.66). This subgroup of patients also completed the* Fatigue Severity Scale (FSS)* showing a statistically significant improvement (*p* < .001). The 26% decrease in average* FSS *scores represents a large effect (*r *= 0.59).

## 5. Discussion

The positive changes reported by UAIHC patients after twelve months of care suggest the potential positive impact of integrative medicine approaches on patient outcomes, including quality of life, work productivity, and well-being.

The SF-12 results showed significant improvement in both physical and mental functioning for patients receiving care in the IM primary care clinic for one year. This result is especially dramatic given patients entered the clinic with mean scores already in the average range compared to the US general population norms of 50 (SD = 10) for physical and mental functioning [22]. Based on the standardized effect sizes for physical (0.20) and mental (0.29) functioning, their 2.2 and 3.1 point changes, respectively, are considered to exceed the minimal clinically important difference [23, 24].

Other studies assessing IM approaches have shown similar improvements with the SF-12. A 6-month, randomized, prospective study of an IM approach to asthma management (incorporating nutrition, yoga, journaling, and nutritional supplements) found a statistically significant improvement in the physical and mental summary scores when compared to a control group [25].

Another IM clinic studied patient reported outcomes following 6 months of treatment for patients with a variety of conditions [26]. The SF-12 was completed by 113 new clinic patients and showed that both mental and physical functioning significantly improved over the 6-month study period.

The SF-12 was also completed by 145 patients with chronic pain, participating in a 6-month evaluation of an IM treatment approach [27]. Results demonstrated significant improvements in physical and mental functioning, similar to those in the present study. It is noteworthy that our study patients experienced similar improvements in quality of life as those with chronic pain, who had lower baseline scores, and therefore, greater likelihood of improving (mental component summary 43.5 vs our 47.2; physical component summary 37.7 vs our 45.4). This seems to indicate that IM primary care has a meaningful impact on patient quality of life even for those who are in alignment with national norms.

Another outcome measure assessing mental functioning found similar results. Patients' psychological well-being improved 12% as measured by the WHO-5, which exceeds the 10% considered to be a clinically meaningful improvement [28, 29]. The baseline score of 55.6 indicates a relatively healthy group; patients with chronic diseases typically score lower: oncology patients 52.2 [30]; diabetes patients 48.4 [31]; depression patients 23.6 [32].

The predictive validity of the WHO-5 has been investigated in a study in which patients with cardiac disease were followed over a period of 6 years [33]. Patients who scored <50 on the WHO-5 at baseline proved to have significantly higher mortality rates compared to those scoring ≥50.

Anxiety and depression scales were another indicator of mental health. Patients experienced a 29% significant improvement in both anxiety (GAD-2) and depression (PHQ-2) measures despite baseline scores well below the threshold indicating need for further evaluation [17, 18].

Patients' perceived stress rated over the past month on the* PSS-4* was the only mental health measure that did not show a significant difference, with only 8% improvement. This four-item measure is an abbreviated form of the original ten-item version (PSS-10). The shorter version may not be as sensitive to detect changes, as indicated by a study of a four-week mindfulness-based stress reduction class given to 23 inner-city patients; despite their 26% improvement in PSS-4 scores, the change was not statistically different [34]. However, when asked to reflect upon the past year using a similar brief measure, patients in the current study perceived an overall improvement in their amount and ability to handle stress.

Similar to the improvement seen in the SF-12 physical summary score, other measures related to physical domains also improved. Pain scores improved 37% which exceeds the 33% standard generally considered a meaningful change from a patient's perspective [35].

Patients in this study did not experience considerable fatigue, but still reported significant improvement with UAIHC. When used as a screening tool, scores of 5 or greater on the Fatigue VAS indicate need for further evaluation [16]. Thus, the 36% improvement in average scores over the year from 5.9 to 3.8 represents a meaningful reduction in risk status in UAIHC patients. In addition, a 26% improvement in fatigue was seen based on the FSS (4.3 to 3.2, p < .001). FSS scores range from 0 to 63, with scores 45 and above indicating significant fatigue [36].

Although it is widely recognized that improvements in diet and exercise are linked with multiple positive impacts on health, we found no similar studies with which to compare our improved BRFSS diet and activity scores.

Perhaps spanning both mental and physical health, there was a 21% statistical improvement in global sleep quality as measured by the PSQI. Findings were similar to results from a 10-week yoga program to reduce menopausal symptoms, which also demonstrated a significant improvement in the global rating of sleep quality [37].

The improvements in both physical and mental well-being for patients receiving IM primary care appear to carry over to the workplace. The WPAI showed improvement in presenteeism (productivity while at work), work impairment, and activity impairment. Although the 38% decrease in absenteeism was not statistically significant, change is difficult to detect given the floor effect observed in this sample (i.e., baseline started very low). Despite the lack of statistical significance, the improvement may have been clinically important to these patients. Studies using the WPAI have found that a 7% improvement in scores led to a meaningful change in patients with Crohn's disease (Sandborn 2007) and a 20% improvement in work productivity or activity impairment represented a clinically meaningful change in patients with psoriasis (Wu 2019).

The lack of statistically significant improvement on the WPAI in our study is not unexpected as many studies have similar results, even in populations where absenteeism would be expected to improve. Patients with depression have substantial work impairment, missing an average 27 days per year due to absenteeism and presenteeism [38]. A study of 548 patients with moderate depression found treated patients (receiving desvenlafaxine 50 mg/d) had significant improvements compared to placebo in the same 3 WPAI domains showing significant improvement in the present study (presenteeism, work impairment, and activity impairment [39]). Looking at the pre-post change scores for the depression treatment group over 26 weeks (as opposed to changes compared to placebo), our study demonstrated larger changes in all 4 WPAI domains.

Other IM modalities have demonstrated improvement in the WPAI as well. A prospective, nonrandomized, open-label observational study evaluated an integrative approach to managing chronic pain [27]. The WPAI survey was completed by 145 participants during the six-month study and all four domains showed statistically significant improvement. The change scores were slightly higher in these patients experiencing chronic pain than the patients receiving care at the UAIHC. This may be explained by the fact that these patients with pain scored worse at baseline on all WPAI measures than UAIHC patients and therefore had greater opportunity for improvement.

### 5.1. Limitations

These results must be considered in light of study limitations. First, outcomes were assessed for the patient population as a whole, which included any patients with available information on any measures at both time points irrespective of presenting problems or consequent treatments. As indicated by the baseline SF-12 and WHO-5, the patients in this study were considered relatively healthy. Thus, results may* underestimate* the improvements on some measures that are most relevant to subgroups by conditions or treatment areas. IM primary care clinics comprised of less healthy patients than our study population would be expected to see even greater improvements in these outcomes measures.

Although participants served as their own controls between baseline and follow-up, real-world observational studies (nonrandomized; nonblinded) cannot exclude possible confounding and selection bias. Further research is needed to clearly delineate the impact of IM primary care on patient-reported outcomes.

The relationship between intensity of care and improvement in patient-reported outcomes was not evaluated. Further research is needed to determine whether numbers of visits or specific provider types contribute to measured outcomes.

Several analyses outlined in our protocol could not be completed due to smaller than anticipated sample size. For example, we planned subgroup analyses for patients with the following five conditions: low back pain, fibromyalgia, other musculoskeletal conditions, metabolic syndrome and diabetes, and cardiovascular disease, assuming IM primary care would have the greatest impact on outcomes in these patients. Similarly, subgroup analyses by intervention or provider types were not feasible. Future research is also needed to explore predictive modeling to identify demographic or health-related conditions at baseline that predict who would benefit most from IM primary care. Examining correlations among various outcomes such as depression and stress would be another future topic to explore with a larger number of patients. Finally, survey measures that ask patients to reflect on the past (e.g., day, week, month, and year) potentially introduce an inherent risk of recall bias.

### 5.2. Conclusions

Following one year of integrative primary care at UAIHC, patient-reported outcomes indicated positive impacts in patients' mental, physical, and overall health; work productivity and activity; and overall well-being. These findings are consistent with other integrative medicine studies that demonstrated enhanced patient well-being. Having focused our analysis on a relatively healthy patient population, we potentially underestimated improvements on some measures that are more clinically relevant to certain patient subgroups. These results are encouraging at a time when there is agreement about the need for more patient-centered models of health care delivery and speaks to the importance and timeliness of the IMPACT study and its contribution to the literature on IM outcomes.

Real-world research is certainly messy. In this study, there was no rigidly defined intervention. Instead, each patient's care and treatment plan was based not only on their clinical history, but also on their personal goals, beliefs and values. In addition, these patients were encouraged to become actively involved in the design of their treatment plan. This principle is at the heart of integrative medicine.

The UAIHC model was designed in the spirit of patient-centeredness, and patient-reported standardized measures were used to evaluate the model's impact. We encourage other researchers to continue assessing the impact of IM on patients as there is both real need and opportunity.

## Figures and Tables

**Table 1:** Summary of patient-reported outcome measures used in this study.

Measure	
*Short Form Health Survey 12-Item (SF-12)*	
	Health-related quality of life measure comprising 12 items that yield overall General Health, Physical and Mental Component summary scores. General Health scores range from 0 (Poor) to 100 (Excellent). Physical and Mental Component Summary scores have an average of 50 and standard deviation of 10 in the general U.S. population. These scores are primarily based on the past 4 weeks. [9]
*Perceived Stress Scale (PSS-4)*	
	Four items yield a summary score of 0 to 16 – with higher scores indicating higher amount of perceived stress – considered representative of the general US population. Respondents are instructed to answer based on past month (4 weeks). [10, 11]
*Stress Response and Amount*	
	Two-item measure with combined ratings ranging from 0 to 12. Higher scores indicate higher stress or inability to handle stress. *Stress amount *item based on past year. [12]
*Work Productivity & Activity Impairment (WPAI)*	
	Seven item-measure of the percent of work productivity loss and impairment in daily activities over the past seven days due to health. WPAI examines two types of work productivity loss among employed respondents: absenteeism, which concerns the amount of time missed from work due to health reasons; and presenteeism, which concerns the amount of impairment while at work (i.e., reduced productivity) due to illness or other health related issues (e.g., pain, fatigue). The WPAI also combines absenteeism and presenteeism to provide an overall metric of work impairment. Impairment in daily activities is also measured separately for all respondents regardless of employment status. [13]
*World Health Organization Well-Being Index (WHO-5)*	
	Five-item measure of emotional well-being yielding an overall percentage score of 0 (worst possible) to 100 (best possible) based on past 2 weeks. Higher scores indicate higher well-being. [14]
*Pain Visual Analog Scale (VAS)*	
	Rated 0 = “No problem” to 10 = “Worst possible problem” based on today. [15]
*Fatigue Visual Analog Scale (VAS)*	
	Rated 0 = “No problem” to 10 = “Worst possible problem” based on past week.
*Fatigue Severity Scale (FSS)*	
	Completed if Fatigue VAS score is 5 or greater Nine item rating summary containing response options ranging from 1 (“Strongly Disagree”) to 7 (“Strongly Agree”) based on the past week. [16]
*Generalized Anxiety Disorder 2 (GAD-2)*	
	Two item rating summary from 0 to 6 based on the past two weeks, with scores > 3 indicating need for further diagnostic evaluation. [17]
*Patient Health Questionnaire 2 (PHQ-2 depression)*	
	Two item rating summary from 0 to 6 based on the past two weeks, with scores > 3 indicating need for further diagnostic evaluation. [18]
*Global Rating of Sleep Quality (Pittsburgh Sleep Quality Index [PSQI] item #9)*	
	One item rating summary from 0 to 3 based on past month, with scores > 2 indicating need for further evaluation. [19]
*Behavioral Risk Factor Surveillance System (BRFSS)*	
	Five items assessing fruit and vegetable intake and nine items measuring exercise and physical activity based on past 30 days. [20]

**Table 2:** UAIHC patient demographics (N = 177).

		*n*	%
Sex			
	Female	123	69.5
	Male	44	24.9
	Missing	10	5.6
Highest level of education completed			
	Completed high school or GED (high school graduate)	4	2.3
	Some college, but have not completed a degree	11	6.2
	Two-year college degree / A.A / A.S.	8	4.5
	Four-year college degree / B.A. / B.S.	63	35.6
	Some graduate work but have not completed a degree	24	13.6
	Completed a Master's degree or professional degree (e.g., ARNP)	51	28.8
	Completed a Ph.D., law degree, M.D., or similar advanced professional degree	15	8.5
	Missing	1	0.6
Employment status			
	Employed full time	117	66.1
	Employed part time	15	8.5
	Unemployed / Looking for work	8	4.5
	Homemaker	12	6.8
	Retired	23	13.0
	Missing	2	1.1
Employer contributes to UAIHC membership fee			
	Yes	69	39.0
	No	102	57.6
	Don't know/Unsure	4	2.3
	Missing	2	1.1
Race/Ethnicity (self-reported)			
	American Indian / Native American / Alaska Native	2	1.1
	Asian or Asian American	1	0.6
	Black / African American	8	4.5
	Hispanic / Latino	8	4.5
	White	153	86.4
	Others	4	2.3
	Missing	1	0.6
Marital Status			
	Married	107	60.5
	Domestic partners	15	8.5
	Divorced	20	11.3
	Widow/widower	5	2.8
	Separated	1	0.6
	Single/Never been married	27	15.3
	Missing	2	1.1
Gross family income from all sources			
	Under $25,000	2	1.1
	$25,000 - $39,999	9	5.1
	$40,000 - $49,999	9	5.1
	$50,000 - $74,999	22	12.4
	$75,000 - $99,999	35	19.8
	$100,000 - $124,999	25	14.1
	$125,000 - $149,999	18	10.2
	Over $150,000	49	27.7
	Missing	8	4.5
Reasons for seeking healthcare at UAIHC			
	To maintain my current level of health	21	11.9
	To prevent a serious health problem in the future	60	33.9
	To maximize my health whether my illness is curable or not	41	23.2
	To use CAM services along with regular health care	25	14.1
	Maximize health (no mention of illness as above)	9	5.1
	All reasons - maintain, prevent, maximize, integrate care	6	3.4
	Address a specific medical issue	9	5.1
	All natural care	1	0.6
	Others	4	2.3
	Missing	1	0.6

**Table 3:** Patient reported outcomes at baseline and 12-month follow-up.

		*n*	Baseline		12 Months		%	
			*Mean*	(SD)	*Mean*	(SD)	Change	
Short Form Health Survey 12-Item (SF-12)								
	Health in general	175	63.9	(23.2)	68.9	(22.5)	8%	*∗∗*
	Physical Component Summary	165	45.4	(10.8)	47.6	(10.1)	5%	*∗∗*
	Mental Component Summary	165	47.2	(10.6)	50.3	(9.3)	7%	*∗∗∗*
Perceived Stress Scale (PSS-4) summary (0-16)		165	4.8	(3.1)	4.36	(2.9)	-8%	
Stress response and amount summary (0-12)		156	7.3	(2.1)	6.5	(2.0)	-10%	*∗∗*
Work Productivity & Activity Impairment (WPAI)								
	% Absenteeism (employed)	101	5.1	(13.5)	3.2	(11.2)	-38%	
	% Presenteeism (employed)	101	19.5	(20.9)	14.1	(20.0)	-28%	*∗∗*
	% Overall work impairment (employed)	100	22.6	(24.0)	15.7	(22.3)	-31%	*∗∗*
	% activity impairment due to health (whether or not employed)	162	26.0	(26.4)	22.0	(27.5)	-16%	*∗*
WHO-5 Overall well-being (0 to 100)		165	55.6	(19.7)	62.2	(19.6)	12%	*∗∗*
Pain Visual Analog Scale (*p*VAS)		65	3.8	(2.2)	2.4	(2.2)	-37%	*∗∗*
Fatigue Visual Analog Scale (*f*VAS)		94	5.9	(1.8)	3.8	(2.3)	-36%	*∗∗*
Fatigue Severity Scale (FSS)		93	4.3	(1.4)	3.2	(1.4)	-26%	*∗∗∗*
Generalized Anxiety Disorder 2 (GAD-2)		158	1.6	(1.6)	1.1	(1.2)	-28%	*∗∗*
Patient Health Questionnaire 2 (PHQ-2 depression)		158	1.2	(1.5)	0.8	(1.3)	-29%	*∗∗*
Global Rating of Sleep Quality (PSQI Item #9)		127	1.5	(0.7)	1.2	(0.7)	-21%	*∗∗∗*
Diet Quality (BRFSS)								
	Fruits: Median # consumed daily	150	1.4	--	1.4	--	-1%	
	Vegetables: Median # consumed daily	155	2.6	--	3.0	--	15%	*∗∗*
Exercise & Physical Activity (BRFSS)								
	Primary activity (# times weekly)	126	3.6	(2.5)	4.3	(3.0)	18%	*∗∗*
	Second activity (# times weekly)	87	2.4	(2.0)	2.9	(3.3)	19%	
	Muscle strengthening (# times weekly)	133	1.4	(1.6)	1.7	(2.1)	26%	*∗*

*∗* p < .05 *∗∗* p < .01 *∗∗∗* p < .001

*Note. *Although means and standard deviations are reported for consistency in this table (except for BRFSS), *p *values are based on paired *t *tests or the nonparametric alternative, *Wilcoxon signed ranks *test, where appropriate.

## Data Availability

The data used to support the findings of this study are included within the article.
